# Contemporary options and future perspectives: three examples highlighting the challenges in testicular cancer imaging

**DOI:** 10.1007/s00345-021-03856-6

**Published:** 2021-11-15

**Authors:** Gamal Anton Wakileh, Christian Ruf, Axel Heidenreich, Klaus-Peter Dieckmann, Catharina Lisson, Vikas Prasad, Christian Bolenz, Friedemann Zengerling

**Affiliations:** 1 Department of Urology and Paediatric Urology, University Hospital Ulm, Albert-Einstein-Allee 23, 89081 Ulm, Germany; 2 Department of Urology, Armed Forces Hospital Ulm, Ulm, Germany; 3 Department of Urology, Uro-Oncology, Robot-Assisted and Specialized Urologic Surgery, Cologne University Hospital, Cologne, Germany; 4 Department of Urology, Asklepios Klinik Altona, Hamburg, Germany; 5 Department for Diagnostic and Interventional Radiology, University Hospital Ulm, Ulm, Germany; 6 Department of Nuclear Medicine, University Hospital Ulm, Ulm, Germany; 7 Surgical Oncology Ulm, i2SOUL Consortium, Ulm, Germany

**Keywords:** Testicular cancer, Germ cell tumors, Seminoma, Non-seminoma, Imaging, Small testicular masses, Staging

## Abstract

**Purpose:**

One of the main issues in testicular germ cell tumors (TGCTs) management is to reduce the necessary amount of treatment to achieve cure. Excess treatment burden may arise from late diagnosis of the primary as well as from false positive or negative staging results. Correct imaging is of paramount importance for successful management of TGCT. The aim of this review is to point out the current state of the art as well as innovative developments in TGCT imaging on the basis of three common challenging clinical situations.

**Methods:**

A selective literature search was performed in PubMed, Medline as well as in recent conference proceedings.

**Results:**

Regarding small testicular lesions, recent studies using elastography, contrast-enhanced ultrasound or magnetic resonance imaging (MRI) showed promising data for differentiation between benign and malignant histology. For borderline enlarged lymph nodes FDG-PET-CT performance is unsatisfactory, promising new techniques as lymphotropic nanoparticle-enhanced MRI is the subject of research in this field. Regarding the assessment of postchemotherapeutic residual masses, the use of conventional computerized tomography (CT) together with serum tumor markers is still the standard of care. To avoid overtreatment in this setting, new imaging modalities like diffusion-weighted MRI and radiomics are currently under investigation. For follow-up of clinical stage I TGCTs, the use of MRI is non-inferior to CT while omitting radiation exposure.

**Conclusion:**

Further efforts should be made to refine imaging for TGCT patients, which is of high relevance for the guidance of treatment decisions as well as the associated treatment burdens and oncological outcomes.

**Supplementary Information:**

The online version contains supplementary material available at 10.1007/s00345-021-03856-6.

## Introduction

Testicular germ cell tumors (TGCTs) represent the most common malignancy among young men aged 15–40 years [1, 2].

Clinically, TGCTs are categorized into seminomas and non-seminomas with relatively equal proportions of 50%–60% for seminomas and 40%–50% for non-seminomas [2, 3]. Among the entire histologic spectrum of testicular neoplasms, TGCTs comprise of 90%–95% of the cases [4, 5] and the remainder comprise of a great variety non-germ cell tumors, for example sex-cord stromal tumors (Leydig cell tumors, Sertoli cell tumors), lymphomas or metastatic lesions arising from other solid malignancies [4].

Clinical diagnosis of TGCTs relies on physical examination, testicular ultrasound and determination of specific tumor markers such as alpha feto protein (AFP), beta-hCG (β-hCG) and lactate dehydrogenase (LDH) [4, 6]. At initial presentation, the tumor is confined to the testis in 68%–75% of the cases (clinical Stage I), regional lymph nodes metastasis in 15%–20% of the cases (clinical Stage II) and distant metastasis in 5%–12% of the cases (clinical Stage III) [6–9].

Currently, computed tomography (CT) is the standard imaging modality for initial staging, recommended by national and international TGCT treatment guidelines [5, 10–12].

Due to the young age of the patient and annual CT imaging after initial therapy, the iatrogenic radiation exposure is a major concern. Depending on the clinical stage, an average of two CT scans per year are performed during a median follow-up time of 4–6 years [13]. This leads to a cumulative increase in radiation exposure over time, which further increases the lifetime risk of second malignancies up to 1.9%–2.6% [13, 14].

In this review, we aim to point out the present best standard as well as future options of improving TGCT management on the basis of three particular clinical scenarios.

## Methods

A selective literature search was performed in PUBMED, Medline as well as in different conference proceedings (ASCO, ESMO, EAU meeting).

## Results

### The ambiguous primary–small testicular lesions

Clinically, the suspicion of a testicular tumor is raised by a painless increase in size of the testis or a hard nodule on palpation. According to current guidelines, scrotal ultrasound is required to confirm the clinical diagnosis and to aid decision making for surgery. Grayscale scrotal sonography is performed with B-mode high frequency (≥ 10 MHz) ultrasound probes providing high resolution images of the scrotal contents. Application of color-coded duplex sonography is used to assess the vascularization of intratesticular masses.

On B-mode ultrasound, seminomas present mostly hypoechogenic, sometimes lobulated and tend to be homogeneous compared to normal testicular tissue [15, 16]. In contrast, most non-seminomas are rather inhomogeneous and show sometimes cystic structures and/or calcifications, which can be explained by more common tumor necrosis, mixed histologies and teratoma components [15, 16]. Despite some distinct differences between seminomas and non-seminomas on ultrasound, no clear differentiation can be made preoperatively.

The widespread use of ultrasound, particularly in the diagnostic workup of infertile men has brought about an increase of incidentally detected small testicular masses [17]. A small testicular mass is usually characterized as a lesion smaller than < 1 cm to < 2 cm, although definitions are conflicting among studies [5, 18, 19]. Recent investigations revealed that about 66%–75% of small masses are benign, mostly consisting of Leydig cell tumors, and other non-germ cell neoplasms that would be overtreated by orchiectomy [20, 21].

**Fig. 1:**
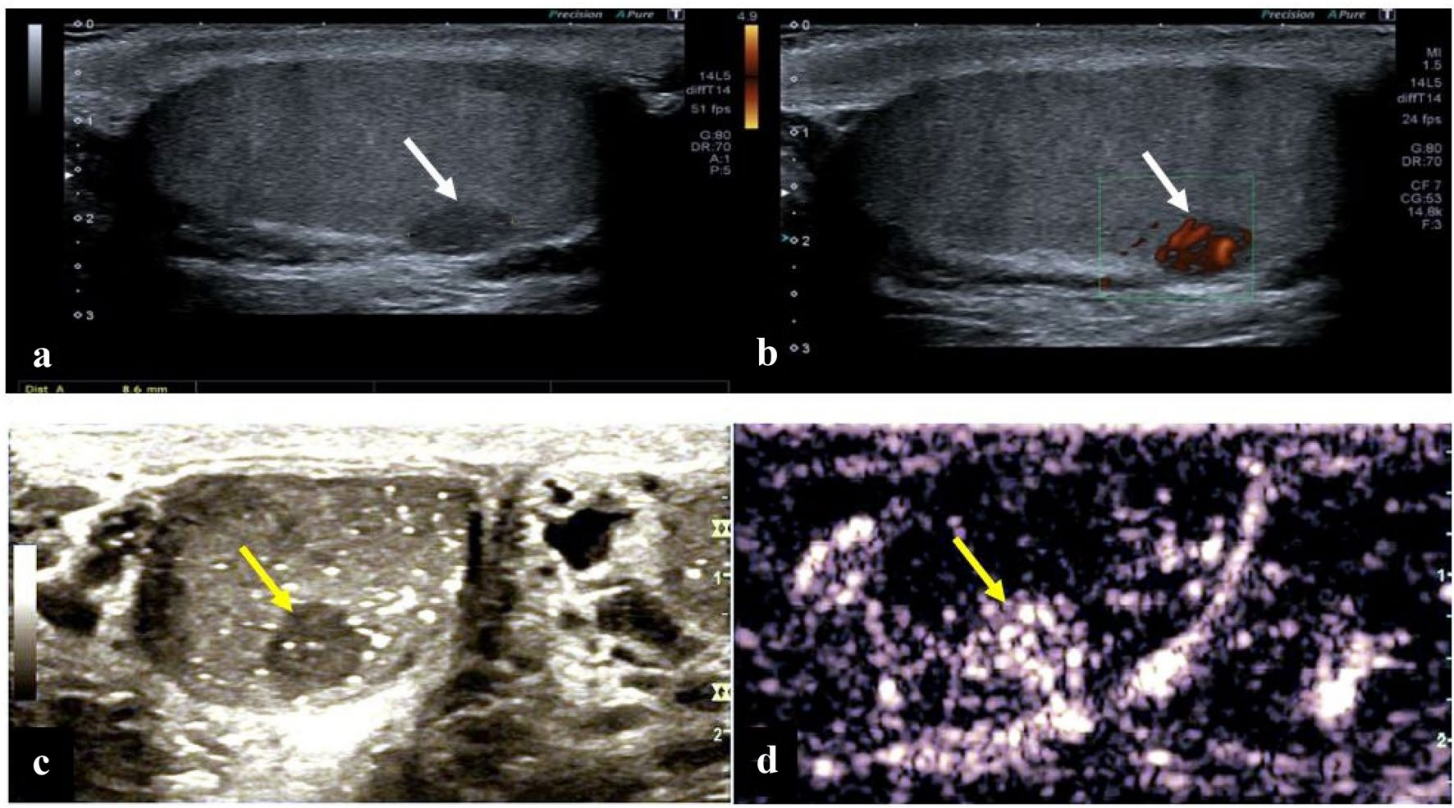
B-mode with Doppler and CEUS ultrasound in small testicular lesions. Case 1(Fig. 1a and 1b): Testicular B-mode grayscale ultrasound with a small homogenous, hypoechogenic lesion (**a**, white arrow) in an 28 year old patient, (**b**) hypervascularization of this lesion (white arrow) in Doppler-Mode. Inguinal exploration confirmed seminoma histology. Case 2(Fig. 1c and 1d): Testicular B-mode grayscale ultrasound with an homogenous lesion and microlithiasis (**c**, yellow arrow) and CEUS with early contrast enhancement in after intravenous contrast agent administration (**d**, yellow arrow). (Pictures c and d are depicted from: “CEUS–use in testicular pathologies”, J. Macron, M. Trottmann, CG Stief, DA Clevert; Der Radiologe. 2018 Jun. 58(6):572–578. Springer-Verlag)

To improve diagnostic accuracy, new ultrasound techniques such as shear wave elastography (SWE) and contrast-enhanced ultrasound (CEUS) have been investigated. Recent studies showed that in SWE an increased stiffness of the suspicious tissue compared to normal testicular tissue can be associated with malignancy [22, 23]. Contrast-enhanced ultrasound (CEUS) versus unenhanced B-mode ultrasound was investigated for the detection of small, nonpalpable testicular tumors < 1.5 cm and for the differentiation between benign and malignant. This technique employs contrast medium that is administered intravenously in the form of “micro gas bubbles”. The oscillation of these microbubbles is detected on ultrasound and allows differentiation between various tissues by specific flooding characteristics (“wash in” and “wash out”) within the tissue. The investigators were able to show that malignant testicular tumors have an increased blood flow and contrast uptake compared to normal testicular parenchyma with a high sensitivity and specifity of 81.6% and 90%. The results are limited by the dependence of the qualitative analysis on the place and region of interest (ROI) and the experience of the observer as well as the lack of external validation of the study results [19]. However, due to the promising results as well as the absence of radiation exposure, this technique can be used as a supportive examination, especially in testicular lesions < 1.5 cm. It was shown that Leydig cell tumors had a faster wash in and prolonged wash out compared to seminomas, which can be used for differential diagnosis. According to the ESUMB guidelines, CEUS ultrasound can be used to visualize and differentiate between vascularized and non-vascularized testicular lesions to help exclude malignancy [24] (Fig. 1).

Scrotal MRI has been investigated as another technique to improve the characterization of equivocal testicular masses [25]. New analytic methods from the MRI sequences, such as histogram, radiomics and diffusion-weighted imaging (DWI) have shown good results in distinguishing between seminomas and non-seminomas [26–30]. Feliciani et al. were able to determine the histology of testicular tumors with an overall accuracy of 86%–89% via MRI radiomics signature [29]. The results are limited by the small number of the assessed lesions (*n* = 44) as well as the lack of external validation and should therefore be considered rather as a proof of concept. Another retrospective work on 41 patients with histologically confirmed germ cell tumors revealed a very good differentiation between seminomas and non-seminomas via DWI-MRI [30]. As overall evidence for MRI to differentiate between malignant and benign testicular tumors is sparse, it is so far only recommended as an auxiliary examination method in ambiguous cases of small intratesticular lesions, doubtful paratesticular masses or in preparation for organ-preserving surgery [5, 25].

In summary, the new ultrasound and MRI techniques mentioned above are able to discriminate benign and malignant testicular lesions with an accuracy not greater than 90%. Therefore, due to the current insufficient data, surgical exploration with subsequent histologic examination is still indispensable [5]. In this context, intraoperative frozen section examination (FSE) is a valuable option during testis surgery and is mandatory in synchronous bilateral tumors, metachronous contralateral tumors or in patients with a solitary testis according to the EAU guidelines [5].

### Detection of retroperitoneal lymph node involvement during initial staging and follow-up of stage I TGCTs

Correct staging and risk stratification is of paramount importance for the management of TGCT patients.

Computed tomography of the chest and abdomen was developed in the early 1980’s and is still the gold standard for staging in TGCT. A recent review by Pierorazio et al. reported contrast-enhanced abdominal CT to have an average sensitivity and specificity of 66.7% (37%–100%) and 95.2% (58%–100%), respectively. The median positive predictive value, negative predictive value and accuracy were shown to be 87.4% (60%–100%), 73.4% (67%–100%) and 83% (71%–100%), respectively [31].

Sensitivity and specificity values for CT are determined by the cut-off applied for lymph node size. Thus, a cut-off for lymph nodes ≥ 10 mm involves a sensitivity of 37% and a specificity of 100% compared to 93% and 58% for a cut-off ≥ 4 mm [31]. A number of investigators tried to improve sensitivity by lowering the threshold for lymph node diameter to be considered pathological. Traditionally, lymph nodes with a short axis diameter of ≥ 10 mm are considered to be pathological [32]. The Swedish Norwegian Testicular Cancer group (SWENOTENCA) defines enlarged lymph nodes larger than 10 mm in long diameter and larger than 8 mm in short diameter, above the aortic bifurcation to be pathological [33]. For non-seminomas, Hilton et al. recommend even lymph nodes equal or larger than 4 mm, especially located anterior to the mid portion of the aorta, to be considered suspicious [34]. In their retrospective analysis, preoperative CT images were correlated with histopathologic results of primary retroperitoneal lymphadenectomy in 70 patients and for a 4 mm threshold a sensitivity of 93% and specificity of 58% was reported [34].

However, the repetitive use of CT, as the gold standard in follow-up care, exposes patients to increased diagnostic radiation. Depending on the clinical stage, TGCT patients receive up to 16 CT scans in a period of 5 years, leading to an increase in the lifetime risk for the development of a second malignancy that ranges between 1.9% and 2.6% [14]. To avoid unnecessary radiation exposure, studies have been conducted comparing MRI with CT in follow-up.

For the assessment of retroperitoneal lymph nodes, abdominal MRI in comparison to abdominal CT yields similar results with a sensitivity between 78% and 96% [35]. The recently published TRISST study, a phase III non-inferiority trial compared abdominal MRI with abdominal CT for detection of early recurrence in patients with clinical stage I seminoma [36]. The study consisted of two study comparisons: 3 MRI scans vs. 3 CT scans as well as 7 MRI scans vs. 7 CT scans. Whereas the proportion of relapses in advanced stages (define as clinical stage ≥ 2C) where similar in both comparisons between CT and MRI, the 3-scan- regimen resulted in a slightly but not significantly higher proportion of relapses in advanced stages (define as clinical stage ≥ 2C) compared to the 7 scan scheme (2.8% vs. 0.3%).

A prospective study on 50 patients with primary TGCTs (seminoma and non-seminoma) investigated the accuracy of DWI-MRI compared to CT for imaging retroperitoneal lymph node metastases. All patients received CT and a subsequent MRI, 30 patients were clinical stage I, 17 were stage II, two were stage III, and one was stage IV. The study showed a sensitivity of 98% for MRI in the detection of retroperitoneal lymph nodes and a non-inferiority compared to CT, but the results are limited by the design, which did not allow the analysis of false-positive results in MRI, due to the low number of control patients [37].

Another large retrospective cohort study investigated abdominal DWI-MRI in the follow-up of clinical stage I TGCTs to detect relapse. A total of 759 patients were included during 2010–2018. Of these, 69% had seminoma (*n* = 524) and 31% had non-seminoma (*n* = 235). DWI-MRI showed a sensitivity of 93.8%, specificity of 97.4%, negative predictive value of 99.7%, positive predictive value of 59.9% and an accuracy of 97.3% in the detection of relapse. With a lymph node cut-off of ≥ 10 mm, the specificity even increased to 100% [38]. For an overview of the performance characteristics of CT and MRT for lymph node imaging according to the literature, see Table S1 in the supplemental material.

Despite its encouraging results, MRI has not yet gained the status of a standard procedure due to limited evidence from further studies, higher costs, longer examination time and the availability of experienced radiologists to interpret the images [31, 32, 35]. From the perspective of the current guidelines, MRI is already recommended in the follow-up of TGCT by the ESMO guideline as well as the German TGCT guideline [10, 11]. Further advances in MRI are awaited from technological innovations, that allow improved differentiation of image sequences such as T1, T2, DWI and as well as the reduction of examination time and costs [38].

Driven by the relatively high false negative rate of traditional imaging methods for clinical stage I, new imaging methods have been investigated during the last decade. Lymphotropic nanoparticle-enhanced MRI (LNMRI) has been tested for TGCT staging [40]. LNMRI is a type of special contrast-enhanced MRI, where ultrasmall superparamagnetic iron oxide (USPIO) such as ferumoxtran-10 is administered intravenously. In a pilot study by Harisinghani, this technique was investigated in 18 patients with stage I testicular cancer. MRI was performed 24 h before intravenous administration and 24 h thereafter. As a control, lymph nodes were obtained per biopsy or surgery and underwent histopathologic examination. The results are very promising as LNMRI had a sensitivity for malignant lymph node involvement of 88.2%, a specificity of 92% and an accuracy of 90.4%. In comparison, the sensitivity of the established size criteria for the detection of malignant nodules on MRI only was 70.5%, the specificity was 68% and the accuracy was 69% [40]. However, the results of the study are limited due to the very small sample size and need confirmation in larger controlled studies.

Currently, whole-body diffusion-weighted MRI imaging, a new enhanced MRI imaging method, is investigated for stage II-III testicular cancer within the prospective TENY study (NCT03436901), after a prospective feasibility study of Mosavi et al., evaluating this technique for follow-up, has reported encouraging results [41, 42].

**Fig. 2:**
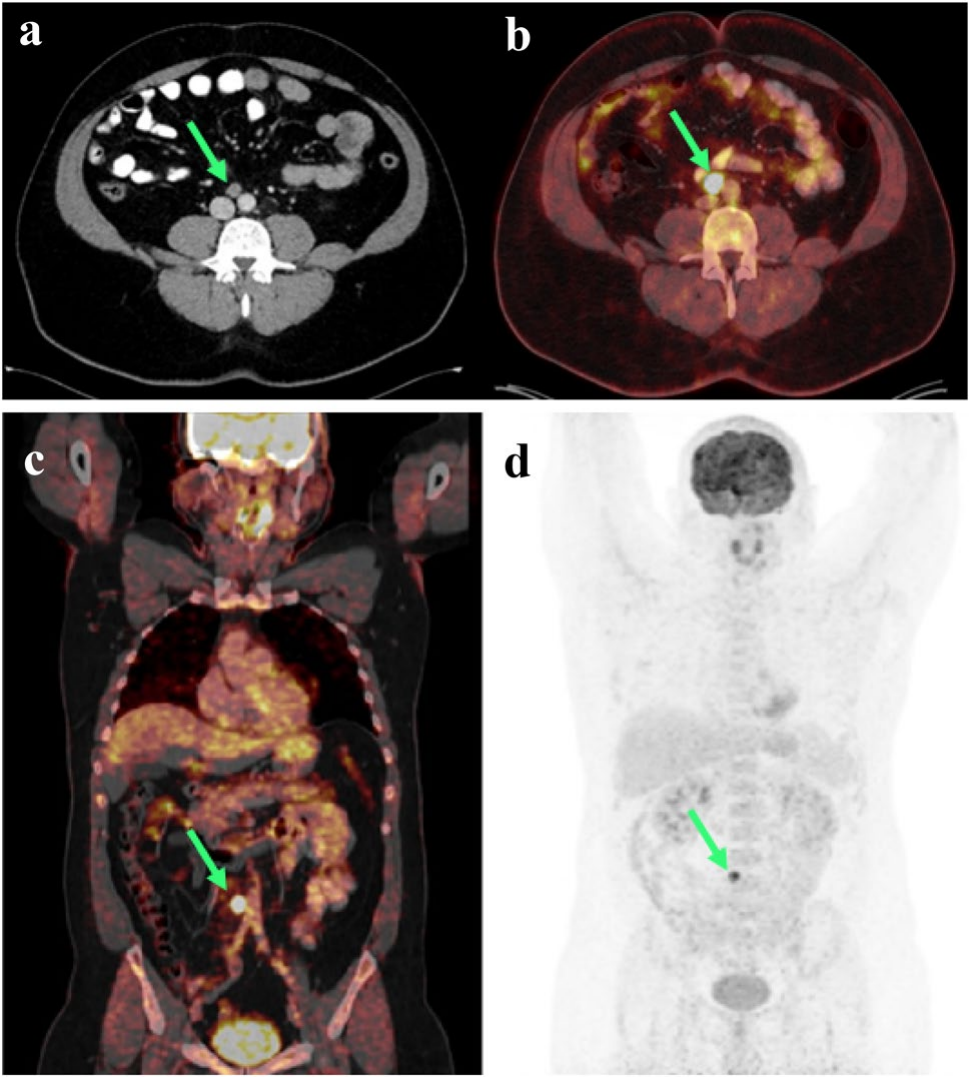
Abdominal and thoracic FDG-PET-CT of an ambiguous retroperitoneal lymph node. **a**: abdominal CT-Scan. Figure **b**–**d**: FGD-PET-CT scan of abdomen/thorax/neck and head with an enlarged interaortocaval lymph node (green arrow) and intense FDG uptake in a patient with right sided seminoma and slightly increased lymph node after orchiectomy (Pictures from “Testicular cancer: Diagnosis and Initial Management”, Springer-Verlag)

A series of studies investigated the value of FDG-PET-CT for initial staging. A recently published review by Pierorazio et al. found for initial staging an overall mean sensitivity, specificity, positive predictive value, negative predictive value, and accuracy for PET-CT of 72% (0%–100%), 90% (0%–100%), 94% (80%–100%), 81% (0%–100%) and 86% (62.5%–100%), respectively [31]. In two studies, focusing on primary retroperitoneal staging of seminomas, the application of a FDG-PET-CT resulted in even better diagnostic values up to 100%, but it did not have a relevant impact on clinical management of these patients [43, 44]. In an older prospective series of 37 stage I–II patients, who underwent retroperitoneal lymph node dissection, FDG-PET-CT failed to identify vital cancer with a diameter less than 5 mm and was therefore judged insufficient to improve staging for early disease stages [45]. In conclusion, because of its limited sensitivity for small metastatic lesions and its excess radiation, the use of FDG-PET-CT is not justified for routine staging or restaging of stage I TGCT patients. However, it can be recommended as an additional tool for inconclusive CT findings [39, 46] (Fig. 2).

### The postchemotherapy residual mass

Approximately 30%–40% of metastatic TGCTs exhibit residual tumors after first-line chemotherapy that consist of necrosis/fibrosis, mature teratomas or viable carcinoma cells in around 40%–50%, 20%–40% and 10–20% of cases [47, 48]. Although PET-CT is currently not of significant value in initial staging, it is recommended in seminomas with residual tumors > 3 cm after first-line chemotherapy or radiotherapy to detect possibly viable tumor cells and to guide further management [5, 47]. The retrospective validation of the SEMPET trial demonstrated for PET-CT after platinum-based chemotherapy in seminoma an overall sensitivity, specificity, negative predictive value, positive predictive value, and accuracy of 67%, 82%, 93%, 42%, and 80%, respectively [49]. The excellent NPV is of particular value since a negative FDG-PET-CT scan allows to keep seminoma patients on follow-up. When FDG-PET-CT was performed after a minimum of 6 weeks after completion of chemotherapy, the sensitivity, specificity, positive predictive value, negative predictive value, and accuracy markedly improved to 82%, 90%, 95%, 69%, and 88%, respectively [49]. However, a recent study challenged these previous findings. Cathomas et al. demonstrated in their retrospective study following 95 patients with metastatic seminoma and residual tumors of > 3 cm after chemotherapy, for FDG-PET-CT an overall positive predictive value of only 23% and a false-positive rate of 77% [50]. In this study, PET results were correlated with histological examination of tissue samples obtained by biopsy or surgery. Thus, of 41 patients with histopathologic review, only 17% had viable seminoma, whereas necrosis was the most frequent finding of PET-positive lesions [50]. Although the majority of the current TGCT guidelines still recommend the use of FDG-PET-CT for postchemotherapeutic seminomas with residual tumors > 3 cm and a minimum 6–8 weeks interval postchemotherapy, the results of Cathomas et. al suggest, that a positive PET-CT scan should not be used as the only parameter for clinical decision making, due to reported high rate of false-positive results [5].

By contrast to seminomas, PET-CT has no role in evaluating the postchemotherapeutic masses of non-seminomas. A prospective trial on 121 patients found no advantage of PET-CT over conventional CT in the imaging of metastatic non-seminomas after chemotherapy [51]. The main reason for this inferior diagnostic performance of PET-CT in nonseminomatous residual tumors is probably the lacking uptake of the PET tracer in tissues with little or metabolic activity such as teratomas [47, 48]. Based on this results,the decision for surgical resection of residual tumors is presently based on conventional contrast-enhanced CT only, with a cut-off of 1 cm [5].

**Fig. 3:**
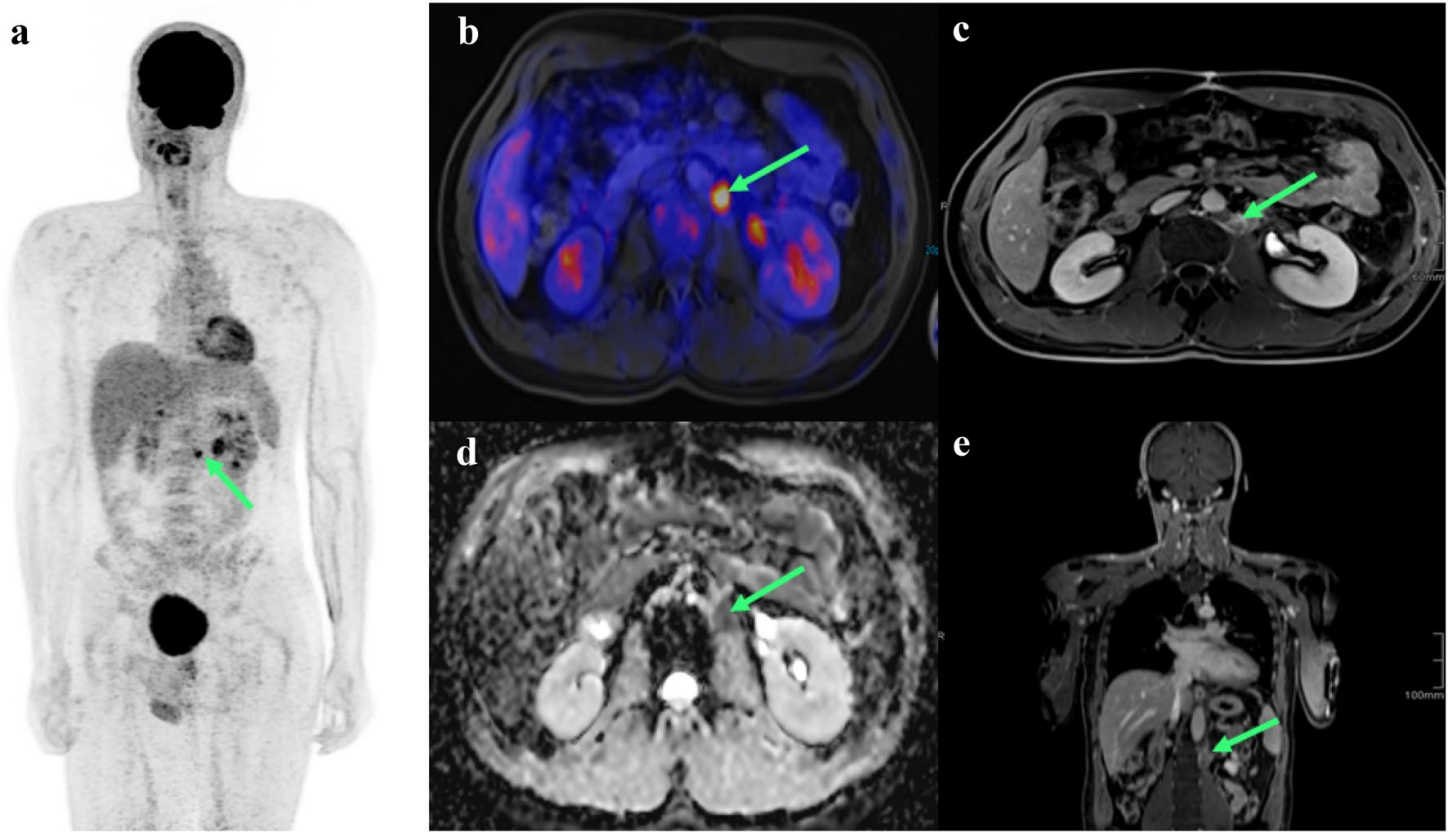
Abdominal FDG-PET/MRI of a retroperitoneal residual tumor in metastatic seminoma after chemotherapy. **A**: Inverted maximum intensity projection (MIP) of an whole-body PET/MRI with a paraaortal lymph node (green arrow). **B**: Transversal image of the abdominal PET/MRI in fusion sequence with high FDG uptake due to increased metabolism (green arrow). **C**: Transversal image of the abdominal PET/MRI in T1 sequence with hyperintens lymph node after contrast agent administration (green arrow). **D**: Transversal image of the abdominal PET/MRI in DWI sequence with hypointens lymph node (green arrow). **E**: Coronar image in T1 sequence with hyperintens lymph node after contrast

Possible improvements in hybrid imaging can either be made by substitution of CT by MRI or by the use of alternative tracers other than FDG. FDG-PET-MRI is an interesting option, that brings together the advantages of PET-based functional imaging with a high soft tissue contrast of MRI technique (Fig. 3/PET-MRT). To our knowledge, it has been investigated only on an individual patient level and study results are yet lacking because of the novelty of the technique.

In terms of new radiotracers, Gallium-68 labeled fibroblast activation protein inhibitor (Ga-68 FAPi), a positron emitter, plays a particular role. Tumor associated activated fibroblasts present a promising target for cancer imaging. An initial study has shown that that Ga-68 FAPi PET shows in comparison to FDG-PET a higher and rather selective tracer uptake in 28 different tumor entities [52]. A phase 1 clinical trial NCT04459273 is underway to assess the role of Ga-68 FAPI PET/CT in several tumor entities including testicular cancers.

Another approach towards improved precision of solid tumor imaging is “radiomics”, an emerging field in radiology, which creates characteristic profiles of tumorous lesions by translating medical images into quantitative data. Recent developments have led to a stronger focus on machine learning and exploring new possibilities in artificial intelligence (AI) modeling [53]. Zheng et al. showed that AI algorithms demonstrated equivalent or even better performance compared to health-care professionals, in terms of sensitivity and specificity when used for the diagnosis of tumor metastasis [54].

Beassler et al. could show that a trained machine learning classifier was able to identify benign posttherapeutic changes in initially affected retroperitoneal lymph node metastases from NSTGCTs with a sensitivity, specificity and accuracy of 88%, 72% and 81%, respectively [55]. Similar results to predict pathology of postchemotherapeutic retroperitoneal lymph node metastases from NSTGCTs with a radiomics approach demonstrated Lewin et al. with a discriminative accuracy of 72% that improved to 88% when combined with clinical predictors [56].

## Conclusion

Imaging is a cornerstone in TGCT management and has high relevance for the guidance of treatment decisions as well as the associated oncological outcomes. New ultrasound and MRI techniques are being introduced into the diagnostic armamentarium to improve the evaluation of unclear small testicular lesions and the clarification of borderline enlarged lymph nodes in patients with known TCGT. MRI should be preferred over CT for abdominal imaging in the follow-up of clinical stage I TGCTs, as it circumvents radiation exposure and recent studies have shown non-inferiority compared with CT. The judgment of postchemotherapeutic retroperitoneal residuals still relies mostly on conventional CT imaging and serum tumor markers, as the advantage of a FDG-PET-CT in this situation has limited benefits and new approaches incorporating radiomics have not yet entered clinical routine.

## Supplementary Information

Below is the link to the electronic supplementary material.

Supplementary file1 (DOCX 22 KB)

## Data Availability

All figures and tables are available without restrictions.

## Declarations

### Human and animals rights

All study protocols were in accordance with the principles of the 1964 Helsinki Declaration and its later amendments.

## References

[1] Bray F, Richiardi L, Ekbom A (2006). Trends in testicular cancer incidence and mortality in 22 European countries: continuing increases in incidence and declines in mortality. Int J Cancer.

[2] Ruf CG, Isbarn H, Wagner W (2014). Changes in epidemiologic features of testicular germ cell cancer: age at diagnosis and relative frequency of seminoma are constantly and significantly increasing. Urol Oncol Semin Orig Investig.

[3] Cheng L, Albers P, Berney DM (2018). Testicular cancer. Nat Rev Dis Primer.

[4] Winter C, Albers P (2011). Testicular germ cell tumors: pathogenesis, diagnosis and treatment. Nat Rev Endocrinol.

[5] Laguna MP, Albers P, Algaba F, et al. (2021) EAU guidelines: testicular cancer. Amsterdam, The Netherlands 2021: EAU guidelines office 2021. https://uroweb.org/guideline/testicular-cancer/ISBN 978-94-92671-13-4. (Accessed 27 Apr 2021)

[6] Rajpert-De Meyts E, McGlynn KA, Okamoto K (2016). Testicular germ cell tumours. Lancet Lond Engl.

[7] Dieckmann K-P, Richter-Simonsen H, Kulejewski M (2018). Testicular germ-cell tumours: a descriptive analysis of clinical characteristics at first presentation. Urol Int.

[8] Horwich A, Shipley J, Huddart R (2006). Testicular germ-cell cancer. Lancet Lond Engl.

[9] SEER Cancer Stat Facts: Testicular Cancer (2018) National cancer institute, bethesda, MD. https://seer.cancer.gov/statfacts/html/testis.html. (Accessed Apr 27, 2021). In: SEER. https://seer.cancer.gov/statfacts/html/testis.html. (Accessed 27 Apr 2021)

[10] Honecker F, Aparicio J, Berney D, et al (2018) ESMO Consensus Conference on testicular germ cell cancer diagnosis treatment and follow-up. Ann Oncol Off J Eur Soc Med Oncol 29:1658–1686. 10.1093/annonc/mdy217 10.1093/annonc/mdy217 30113631

[11] Kliesch S, Schmidt S, Wilborn D (2021). Management of germ cell tumours of the testis in adult patients. german clinical practice guideline part i: epidemiology, classification, diagnosis, prognosis, fertility preservation, and treatment recommendations for localized stages. Urol Int.

[12] Kliesch S, Schmidt S, Wilborn D (2021). Management of germ cell tumours of the testes in adult patients: german clinical practice guideline, PART II–recommendations for the treatment of advanced, recurrent, and refractory disease and extragonadal and sex cord/stromal tumours and for the management of follow-up, toxicity, quality of life, palliative care, and supportive therapy. Urol Int.

[13] Sullivan CJ, Murphy KP, McLaughlin PD (2015). Radiation exposure from diagnostic imaging in young patients with testicular cancer. Eur Radiol.

[14] Tarin TV, Geoffrey S, Rajesh S (2009). Estimating the risk of cancer associated with imaging related radiation during surveillance for stage i testicular cancer using computerized tomography. J Urol.

[15] Marko J, Wolfman DJ, Aubin AL, Sesterhenn IA (2017). Testicular seminoma and its mimics: from the radiologic pathology archives. Radiographics.

[16] Necas M, Muthupalaniappaan M, Barnard C (2021). Ultrasound morphological patterns of testicular tumours, correlation with histopathology. J Med Radiat Sci.

[17] Toren PJ, Roberts M, Lecker I (2010). Small incidentally discovered testicular masses in infertile men–is active surveillance the new standard of care?. J Urol.

[18] Paffenholz P, Held L, Loosen SH (2018). Testis sparing surgery for benign testicular masses: diagnostics and therapeutic approaches. J Urol.

[19] Isidori AM, Pozza C, Gianfrilli D (2014). Differential diagnosis of nonpalpable testicular lesions: qualitative and quantitative contrast-enhanced us of benign and malignant testicular tumors. Radiology.

[20] Giannarini G, Dieckmann K-P, Albers P (2010). Organ-sparing surgery for adult testicular tumours: a systematic review of the literature. Eur Urol.

[21] Bieniek JM, Juvet T, Margolis M (2018). Prevalence and management of incidental small testicular masses discovered on ultrasonographic evaluation of male infertility. J Urol.

[22] Rocher L, Criton A, Gennisson J-L (2019). Characterization of testicular masses in adults: performance of combined quantitative shear wave elastography and conventional ultrasound. Ultrasound Med Biol.

[23] Pedersen MR, Møller H, Osther PJS (2017). Comparison of tissue stiffness using shear wave elastography in men with normal testicular tissue, testicular microlithiasis and testicular cancer. Ultrasound Int Open.

[24] Sidhu PS, Cantisani V, Dietrich CF (2018). The EFSUMB guidelines and recommendations for the clinical practice of contrast-enhanced ultrasound (CEUS) in non-hepatic applications: Update 2017 (Long Version). Ultraschall Med-Eur J Ultrasound.

[25] Tsili AC, Bertolotto M, Turgut AT (2018). MRI of the scrotum: recommendations of the ESUR scrotal and penile imaging working group. Eur Radiol.

[26] Zhang P, Min X, Feng Z (2021). Value of intra-perinodular textural transition features from MRI in distinguishing between benign and malignant testicular lesions. Cancer Manag Res.

[27] Min X, Feng Z, Wang L (2018). Characterization of testicular germ cell tumors: whole-lesion histogram analysis of the apparent diffusion coefficient at 3T. Eur J Radiol.

[28] Zhang P, Feng Z, Cai W (2019). T2-weighted image-based radiomics signature for discriminating between seminomas and nonseminoma. Front Oncol.

[29] Feliciani G, Mellini L, Carnevale A (2021). The potential role of MR based radiomic biomarkers in the characterization of focal testicular lesions. Sci Rep.

[30] Tsili AC, Ntorkou A, Astrakas L (2017). Diffusion-weighted magnetic resonance imaging in the characterization of testicular germ cell neoplasms: effect of ROI methods on apparent diffusion coefficient values and interobserver variability. Eur J Radiol.

[31] Pierorazio PM, Cheaib JG, Tema G (2020). Performance characteristics of clinical staging modalities for early stage testicular germ cell tumors: a systematic review. J Urol.

[32] Kreydin EI, Barrisford GW, Feldman AS, Preston MA (2013). Testicular cancer: what the radiologist needs to know. Am J Roentgenol.

[33] Forsberg L, Dale L, Høiem L (1986). Computed tomography in early stages of testicular carcinoma. Size of normal retroperitoneal lymph nodes and lymph nodes in patients with metastases in stage II A. A SWENOTECA study: Swedish-Norwegian testicular cancer project. Acta Radiol Diagn (Stockh).

[34] Hilton S, Herr HW, Teitcher JB (1997). CT detection of retroperitoneal lymph node metastases in patients with clinical stage I testicular nonseminomatous germ cell cancer: assessment of size and distribution criteria. AJR Am J Roentgenol.

[35] Sohaib SA, Koh DM, Barbachano Y (2009). Prospective assessment of MRI for imaging retroperitoneal metastases from testicular germ cell tumours. Clin Radiol.

[36] Joffe JK, Cafferty FH, Murphy L (2021). Imaging modality and frequency in surveillance of stage I seminoma testicular cancer: results from a randomized, phase III, factorial trial (TRISST). J Clin Oncol.

[37] Laukka M, Mannisto S, Beule A (2020). Comparison between CT and MRI in detection of metastasis of the retroperitoneum in testicular germ cell tumors: a prospective trial. Acta Oncol.

[38] Larsen SKA, Agerbæk M, Jurik AG, Pedersen EM (2020). Ten years of experience with MRI follow-up of testicular cancer stage I: a retrospective study and an MRI protocol with DWI. Acta Oncol.

[39] de Wit M, Brenner W, Hartmann M (2008). [18F]-FDG-PET in clinical stage I/II non-seminomatous germ cell tumours: results of the German multicentre trial. Ann Oncol Off J Eur Soc Med Oncol.

[40] Harisinghani MG, Saksena M, Ross RW (2005). A pilot study of lymphotrophic nanoparticle-enhanced magnetic resonance imaging technique in early stage testicular cancer: a new method for noninvasive lymph node evaluation. Urology.

[41] Larsen SKA (2020) Whole body MRI with DWI for monitoring patients treated for testicular cancer stage II–III ; https://clinicaltrials.gov/ct2/show/NCT03436901. (Accessed 03 May 2021). clinicaltrials.gov

[42] Mosavi F, Laurell A, Ahlström H (2015). Whole body MRI, including diffusion-weighted imaging in follow-up of patients with testicular cancer. Acta Oncol.

[43] Ambrosini V, Zucchini G, Nicolini S (2014). 18F-FDG PET/CT impact on testicular tumours clinical management. Eur J Nucl Med Mol Imaging.

[44] Hain SF, O’Doherty MJ, Timothy AR (2000). Fluorodeoxyglucose PET in the initial staging of germ cell tumours. Eur J Nucl Med.

[45] Albers P, Bender H, Yilmaz H (1999). Positron emission tomography in the clinical staging of patients with stage I and II testicular germ cell tumors. Urology.

[46] Li Y, Jiang L, Wang H (2019). Effective radiation dose of 18F-fdg PET/CT: how much does diagnostic CT contribute?. Radiat Prot Dosimetry.

[47] Daneshmand S, Albers P, Fosså SD (2012). Contemporary management of postchemotherapy testis cancer. Eur Urol.

[48] Pfannenberg AC, Oechsle K, Bokemeyer C (2004). The role of [(18)F] FDG-PET, CT/MRI and tumor marker kinetics in the evaluation of post chemotherapy residual masses in metastatic germ cell tumors–prospects for management. World J Urol.

[49] Bachner M, Loriot Y, Gross-Goupil M (2012). 2–18fluoro-deoxy-D-glucose positron emission tomography (FDG-PET) for postchemotherapy seminoma residual lesions: a retrospective validation of the SEMPET trial. Ann Oncol.

[50] Cathomas R, Klingbiel D, Bernard B (2018). Questioning the value of fluorodeoxyglucose positron emission tomography for residual lesions after chemotherapy for metastatic seminoma: results of an international global germ cell cancer group registry. J Clin Oncol.

[51] Oechsle K, Hartmann M, Brenner W (2008). [18F]Fluorodeoxyglucose positron emission tomography in nonseminomatous germ cell tumors after chemotherapy: the German multicenter positron emission tomography study group. J Clin Oncol Off J Am Soc Clin Oncol.

[52] Kratochwil C, Flechsig P, Lindner T (2019). 68Ga-FAPI PET/CT: tracer uptake in 28 different kinds of cancer. J Nucl Med.

[53] Rogers W, Thulasi Seetha S, Refaee TAG (2020). Radiomics: from qualitative to quantitative imaging. Br J Radiol.

[54] Zheng Q, Yang L, Zeng B (2020). Artificial intelligence performance in detecting tumor metastasis from medical radiology imaging: a systematic review and meta-analysis. EClinicalMedicine.

[55] Baessler B, Nestler T, Pinto Dos Santos D (2020). Radiomics allows for detection of benign and malignant histopathology in patients with metastatic testicular germ cell tumors prior to post-chemotherapy retroperitoneal lymph node dissection. Eur Radiol.

[56] Lewin J, Dufort P, Halankar J (2018). Applying radiomics to predict pathology of postchemotherapy retroperitoneal nodal masses in germ cell tumors. JCO Clin Cancer Inform.

