# Supplementary material for: Contemporary options and future perspectives: three examples highlighting the challenges in testicular cancer imaging
Source: World J Urol. 2021 Nov 15;40(2):307–15. doi: 10.1007/s00345-021-03856-6 (PMC8921012; doi:10.1007/s00345-021-03856-6)
Supplement: Supplementary file 1 — Supplementary file1 (DOCX 22 KB) [file 345_2021_3856_MOESM1_ESM.docx]

**Contemporary options and future perspectives: three examples highlighting the challenges in testicular cancer Imaging**

Gamal A. Wakileh ^1^, Christian Ruf^2^, Axel Heidenreich^3^, Klaus-Peter-Dieckmann^4^, Catharina Lisson^5^, Vikas Prasad^6^, Christian Bolenz^1^, Friedemann Zengerling^1,*^

^1^ Department of Urology and Pediatric Urology, Ulm University Hospital, Ulm, Germany

^2^ Department of Urology, Armed Forces Hospital Ulm, Ulm, Germany

^3^ Department of Urology, Department of Urology, Uro-Oncology, Robot-assisted and Specialized Urologic Surgery, Cologne University Hospital, Cologne, Germany

^4^ Department of Urology, Asklepios Klinik Altona, Hamburg, Germany

^5^ Department for Diagnostic and Interventional Radiology, Ulm University, Ulm, Germany.

^6^ Department of Nuclear Medicine, Ulm University, Ulm, Germany.

Keywords: testicular cancer, germ cell tumors, seminoma, non-seminoma, imaging, small testicular masses, staging,

*Corresponding author:

Friedemann Zengerling, MD

Department of Urology and Paediatric Urology

Ulm University Hospital, Ulm, Germany

Albert-Einstein-Allee 23

89081 Ulm, Germany

Tel.: +49-731- 500-58036

Fax.: +49- 731 500-58002

E-Mail: friedemann.zengerling@uniklinik-ulm.de

| Autor | Image Modality | Patient count (n) | Lymph node size (mm) or histologic subclassification | Sensitivity (%) | Specificity (%) | PPV (%) | NPV (%) | Accuracy (%) |
| --- | --- | --- | --- | --- | --- | --- | --- | --- |
| Forsberg et. al (1986) (SWENOTECA) [33] | CT | n = 156 | 8 + 10 in SD and LD | 34 | 83 | 44 | 76 | 69 |
| Hilton et al. (1997) [34] | CT | n = 70 | ≥ 10 | 37 | 100 | / | / | / |
|  |  |  | ≥ 8 | 47 | 100 | / | / | / |
|  |  |  | ≥ 6 | 67 | 83 | / | / | / |
|  |  |  | ≥ 4 | 93 | 58 | / | / | / |
| De Wit et. al (2008) [39] | CT | n = 72 | > 10  Non-seminoma | 41 | 95 | 87 | 67 | 71 |
|  | | | | | | | | |
| *Sohaib et al. (2009)* [35] | MRI | n = 52 | > 10 | 78-96 | / | / | / | / |
| Laukka et al. (2020) [37] | MRI | n = 50 | ≥ 7 | 98 | / | / | / | / |
| Larsen et al. (2020) [38] | MRI | n = 759 | total  ≥ 10 in SD | 93.8  100 | 97.4 | 59.9 | 99.7 | 97.3 |

Abbreviation: CT= computed tomography; LD = long diameter; mm = millimeter; MRI = magnetic resonance imaging; n = number; NPV: negative predictive value; PPV = positive predictive value; SD = short diameter;

**Supplementary Table S1: Performance characteristics of CT and MRT for abdominal imaging according to the literature**
